# A Visible-Light-Enhanced Heterogeneous Photo Degradation of Tetracycline by a Nano-LaFeO_3_ Catalyst with the Assistance of Persulfate

**DOI:** 10.3390/nano13081388

**Published:** 2023-04-17

**Authors:** Liwei Hou, Yanan Wang, Fan Zhou, Shuangyue Liu, Lin Fu, Lei Wang, Changbo Zhang, Weijie Xue

**Affiliations:** 1 College of Ocean Science and Engineering, Shanghai Maritime University, Shanghai 201306, China; 2 Key Laboratory of Original Agro-Environmental Pollution Prevention and Control, Agro-Environmental Protection Institute, Ministry of Agriculture and Rural Affairs, Tianjin 300191, China; 3 College of Resources and Environment, Northeast Agricultural University, Harbin 150030, China; 4 College of Information Engineering, Shanghai Maritime University, Shanghai 201306, China; 5 Key Laboratory of Environmental Biotechnology, Research Center for Eco-Environmental Sciences, Chinese Academy of Sciences, Beijing 100085, China

**Keywords:** nanoscaled LaFeO_3_, visible light photocatalysis, tetracycline, acute toxicity

## Abstract

Perovskites with nano-flexible texture structures and excellent catalytic properties have attracted considerable attention for persulfate activation in addressing the organic pollutants in water. In this study, highly crystalline nano-sized LaFeO_3_ was synthesized by a non-aqueous benzyl alcohol (BA) route. Under optimal conditions, an 83.9% tetracycline (TC) degradation and 54.3% mineralization were achieved at 120 min by using a coupled persulfate/photocatalytic process. Especially compared to LaFeO_3_-CA (synthesized by a citric acid complexation route), the pseudo-first-order reaction rate constant increased by 1.8 times. We attribute this good degradation performance to the highly specific surface area and small crystallite size of the obtained materials. In this study, we also investigated the effects of some key reaction parameters. Then, the catalyst stability and toxicity tests were also discussed. The surface sulfate radicals were identified as the major reactive species during the oxidation process. This study provided a new insight into nano-constructing a novel perovskite catalyst for the removal of tetracycline in water.

## 1. Introduction

Water contamination by pharmaceuticals has become an environmental issue of concern in recent decades. Most pharmaceuticals were directly excreted into domestic wastewater without effective treatment by conventional water treatment sites. Up until now, various kinds of drugs, such as antibiotics, hormones, and preservatives, have been identified within water environmental systems [1]. Because of their antibacterial natures, the antibiotic residues within contaminated waters cannot be effectively removed by traditional biological methods. The chemical advanced oxidation processes (AOPs) for wastewater treatment appear then as reliable alternatives to biodegradation processes. AOPs involve the generation of hydroxyl radicals (^•^OH) [2,3,4,5]. In addition to hydroxyl-radicals-based reactions, some other AOPs, including peroxydisulfate-activated reactions, ultrasounds, UV-vis irradiation, and ozonation, have received great attention over the last decades [6,7,8,9,10]. In a “hybrid” AOP, consisting of visible light and the activation of peroxysulfate (PS), the sulfate radical was produced from the decomposition of persulfate ions (S_2_O_8_^2−^) by using heat or even transition metal (Equations (1) and (2)) [7]:

S_2_O_8_^2−^ + heat → 2SO_4_^•−^ (1)

S_2_O_8_^2−^ + Me^n+^ →SO_4_^•−^ + SO_4_^2−^ + Me^(n+1)^ (2)

As shown in Equations (3)–(5), in sulfate-radicals-mediated photo-Fenton-like reactions, PS is an oxidant activated by the catalyst to produce reactive radicals (SO_4_^•−^, ^•^OH, and others), since it acts as an electron acceptor that can decrease the recombination between photogenerated electrons (e^−^) and holes (h^+^) [8]. Consequently, h^+^ reacts with PS to generate SO_4_^•−^ (Equation (3)). The formed e^−^ is able to decompose the PS to generate SO_4_^•−^ (Equation (4)). In addition, the PS can be activated in the presence of visible light, generating SO_4_^•−^ (Equation (5)). If a homogeneous ferrous catalyst is used, Fe^2+^ can be regenerated by the photogenerated e^−^. However, the development of more efficient and stable photoactivation catalysts for sulfate-radical photo-Fenton processes is required.

S_2_O_8_^2−^ + h*v*_b_^+^ → 2SO_4_^•−^ (3)

S_2_O_8_^2−^ + e^−^ → 2SO_4_^•−^ (4)

S_2_O_8_^2−^ + h*v* → 2SO_4_^•−^ (5)

Perovskite-based oxides, of the general formula ABO_3_, are important heterogeneous catalysts due to the low cost and flexibility of their composition, given their adjustable redox properties [11]. Therefore, they are especially studied within gas-phase catalytic depollution processes, including oxidation or reduction (CO, methane, VOCs, soot, and NO_x_, etc.) [12,13,14]. Perovskites have been proposed as potential alternatives to the commercially used supported noble metals (mostly based on Pt, Pd formulations) [14]. Among the developed perovskites, iron-based compositions are found to present advantages for catalytic processes, due to their excellent high-temperature thermal and hydrothermal stability, redox activity, and the low cost of their constituting elements [15,16,17,18]. More interesting, in addition to their efficiency for gas-phase effluent treatments, perovskites can also serve as catalysts for wastewater treatment, due to the presence of transition metals with multiple valence states. It is reported, for example, that BiFeO_3_-type perovskites (BFO) are active catalysts for oxidative acetylation [19], photocatalysis [20,21,22,23,24,25,26], hydrogen generation [27], and the catalytic activation of H_2_O_2_ [28]. BiFeO_3_/ZnFe_2_O_4_ nanocomposites have also been employed as catalysts for the removal of azo dyes under visible light irradiation [28].

Owing to its stable crystal structure, excellent optical properties, and narrow bandgap (~2.1 eV), LaFeO_3_ has been considered as a promising photoactive candidate. However, because LaFeO_3_ is a p-type semiconductor, the rapid recombination of e^−^ and h^+^ have been reported [29]. In addition, LaFeO_3_ displays a low activity due to its high charge recombination rates. Therefore, introducing peroxides into the photocatalysis system may be an efficient strategy with the consideration of the capture effect of peroxides for photo-induced electrons on the conduction band of a photocatalyst. In this frame, it could be anticipated that the synergistic effects of the photocatalytic process and sulfate-radical-based performance could exhibit a higher degradation rate for organic pollutants than the Fenton process. As reported, peroxymonosulfate (PMS) was evidenced to be a good candidate for avoiding the recombination of e^−^ and h^+^ and for improving the degradation efficiency of organic compounds [30,31,32,33,34,35]. Compared to H_2_O_2_, the longer O–O bond length and asymmetric structure of HSO_5_^−^ are supposed to guarantee the easier dissociation of PMS over organic compounds. However, some problems still remain, such as the limited catalytic activity of perovskite considering the low surface developed by the perovskites [11], the underutilization of PS [36], and the low stability of the catalyst with iron ions leaching occurring during the reaction [37].

From a fundamental point of view, textural properties, structural properties, and redox properties are interrelated parameters that have a significant effect on the catalytic performance of perovskites. However, using conventional methods for perovskite synthesis, such as calcination milling [17] and even hydroxyacid complexation routes [38,39], lead to a large grain size (D), a low specific surface area (SSA), and a poor activity [24,25]. Indeed, when using a classical complexation approach [38,39], precursor complexation with hydroxyacid allows for the cations to be kept homogeneously dispersed in a hybrid matrix, ensuring crystallization at a lower temperature than that for solid–solid approach, i.e., 600–650 °C. Better catalytic performances are achieved in this last case due to improved textural properties, but an accessible surface area is not the only parameter affecting this catalysis, since the B-cation surface concentration, reducibility, and oxygen mobility of the catalysts are also considered to be key parameters [11,12,26]. Some improved synthetic approaches have then been proposed to obtain higher surface areas (30–100 m^2^ g^−1^) and smaller crystal sizes (<20 nm). Typical examples of these include the sol-gel-derived method [13], reactive grinding [40], nanocasting [41], and flame-spray pyrolysis [42], etc. Reactive grinding is an excellent example that allows for the production of high-surface-area materials (reported to reach 100 m^2^ g^−1^ [43]), but its popularization is restricted due to the requirement of special equipment, and this approach is better adapted to large-scale synthesis than laboratory-scale synthesis [44,45]. However, it is still a real challenge to directly synthesize size-limited and pure nanocrystalline perovskites with particular porosities. A benzyl alcohol (BA) route was proposed as a valuable approach for oxide and mixed oxide nanoparticle preparations. This route not only offers an excellent control over the particle size, shape, crystallinity, order, purity, and assembly behavior, but also avoids the use of any additional surfactants or templates [46,47,48,49]. The BA route is defined as a non-aqueous synthesis, since during the different competitive reactions, no water is added, which affords a better control over the crystal assembly.

In this work, nano-LaFeO_3_ was prepared by a citric acid complexation route and benzyl alcohol route. Using an adapted synthesis procedure [49], the benzyl-alcohol-derived material presented quasi-spherical nanosized particles with a much higher specific surface area, as well as a lower crystal size domain size. The reaction parameters for the sulfate-radical–photo-Fenton tetracycline degradation process were optimized (operating pH, oxidant concentration, and catalyst loading) with this catalyst. The catalyst stability was also evaluated upon the reaction cycling, which was followed by metal ion leaching.

## 2. Materials and Methods

### 2.1. Materials and Methods

The synthesis was performed by a benzyl alcohol route.

The sample LaFeO_3−_BA: La(NO_3_)_3_·6H_2_O (2 mmol) was dissolved in anhydrous benzyl alcohol (26.7 mL) at room temperature. After that, iron (III) acetylacetonate (2 mmol) was added dropwise into the benzyl alcohol (40 mL) at room temperature. The reaction mixture was thereafter stirred for 30 min and transferred into a Teflon-lined autoclave. The autoclave was heated to 200 °C for 24 h. The resulting brown suspension was recovered by centrifugation (4 cycles, with washing with ethanol between each cycle). The centrifugation was performed at 8000 rpm for 10 min. The washed material was dried at 80 °C for 12 h, and the dry solid was calcined at 500 °C for 6 h. The prepared sample was labeled as the BA sample.

The synthesis was then performed by a citric acid complexation route.

The sample LaFeO_3−_CA: aqueous solutions of lanthanum(III) nitrate hexahydrate (20 mM) (LaNO_3_∙6H_2_O ≥ 99.0 %, Sigma-Aldrich, Houston, USA) and iron(III) nitrate hexahydrate (20 m mol L^−1^) (97.7%, Alfa Aesar, Shanghai, China) were prepared with distilled water. Thereafter, an aqueous solution of citric acid (98 %, Prolabo, Paris, France), with a molar equivalence to La + Fe (40 mmol L^−1^), was added dropwise to the precursor solution under gentle stirring at 80 °C. Ageing was performed until a gel was obtained. The gel was thereafter dried in an oven at 110 °C for 12 h. Subsequently, the obtained foam solid was crushed and calcined at 500 °C for 6 h. The sample was labeled as the CA sample.

### 2.2. Experimental

**Figure 1:**
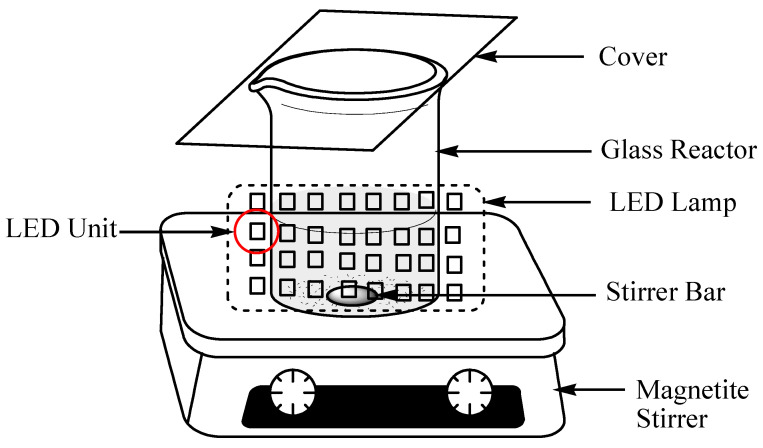
Scheme of the experimental reaction configuration with a LED strip as light source.

The reaction configuration: the experiment was carried out in a photo-reactor consisting of visible LED lamps, as shown in Figure 1. The LED lamp emitted white light at a wavelength of 455 nm and further broadband stokes-shifts were emitted at roughly 500–600 nm.

For the activity measurement, a solution of tetracycline (TC, purity, and source) was prepared with deionized water (in the two hours preceding the reaction). The initial concentration (C_0_) was fixed at 100 mg L^−1^ and the C_0_ exact value was verified by an HPLC analysis. Then, a selected weight of the LaFeO_3_ was added into 200 mL of the TC solution, with constant stirring that was maintained using a mechanical stirrer (RW 20, IKA, Staufen, Germany) for 15 min, in order to achieve adsorption–desorption equilibrium on the solid catalyst surface. At this time, the [TC]_0_ value—the concentration of the TC after the adsorption process—was obtained. Then, a persulfate (PS) solution, prepared by the dissolution of 1 mol L^−1^ (purity and source) in water to obtain a PS concentration of 50 mmol L^−1^, was added to start the reaction. All the experiments were conducted at ambient temperature (22 ± 2 °C). The reaction solution was sampled (1 mL) over fixed intervals and mixed with 1 mL of methanol (0.1 mol L^−1^) for at least 15 min to terminate the reaction. Then, the reaction solution was filtered through a 0.22 μm membrane (Millipore Co., Molsheim, France) and mixed with the same volume of methanol to quench the reaction. The TC conversion (*X_TC_*) and the total organic carbon (TOC) abatement (*X_TOC_*) were calculated by Equations (6) and (7), respectively.

(6) $$X_{TC}=\frac{C_{0}-C}{C_{0}}\times 100\%$$

(7) $$X_{TOC}=\frac{TOC_{0}-TOC}{TOC_{0}}\times 100\%$$

where *C* is the TC concentration at time t (min), *TOC* is the total organic carbon time *t* (min), and *TOC***_0_** is the initial total organic carbon after stabilization due to the adsorption on the catalyst surface.

### 2.3. Characterization and Analysis

X-ray diffraction (XRD) was performed. The X-ray diffraction patterns were obtained on a D5005 diffractometer (Bruker, Karlsruhe, Germany) equipped with Cu Kα radiation (λ = 1.5406 Å). The signal was recorded for 2*θ* between 20° and 80°, with a recording step of 0.05° (3 s per step). A phase identification was performed by a comparison with the JCPDS database. The crystal size was determined using the Scherrer equation after applying Warren’s correction for the instrumental broadening.

For the N_2_-physisorption, N_2_ adsorption measurements were performed on a Micromeritics TRISTAR 3000 instrument. A desorption isotherm was used to determine the pore size distribution using the Barret Joyner Halenda (BJH) method. The specific surface areas (SSA) of the catalysts were obtained by applying the Brunauer–Emmett–Teller (BET) equation (0.10 < P/P_0_ < 0.35). Prior to the analysis, the samples (0.5 g) were heated at 250 °C under a vacuum for 6 h.

The specific surface area (ssa) can then be derived:

(8) $$ssa=\frac{V_{m}N_{A}a_{m}}{v_{m}m_{s}}$$

where *N*_A_ is the Avogadro’s number (6.022 × 10^23^ mol^−1^), *a_m_* is the effective cross-section area of one adsorbed molecule, *v_m_* is the molar volume of one adsorbed molecule (22,400 mL of the volume occupied by 1 mol of the adsorbate gas under standard conditions), and *m_s_* is the mass of the substrate/adsorbent.

Then, transmission electron microscopy (TEM) was used. The catalyst morphology was determined by a TEM analysis coupled with energy-dispersive X-ray spectroscopy (EDXS). The micrographs were collected on a JEM 2100 instrument (JEOL, Tokyo, Japan), operated at 200 kV with a LaB6 source and equipped with a Gatan Ultra scan camera. The EDX spectroscopy was carried out with a Hypernine (Premium, JEOL, Japan) detector (active area: 30 mm^2^), using the SM-JED 2300T software for the data acquisition and treatment.

All the catalysts were systematically characterized using X-ray diffraction (XRD), X-ray photoelectron spectroscopy (XPS), N_2_-physisorption, and ICP-OES (see the Supplementary Data for details).

The tetracycline concentration was determined by using a high-performance liquid chromatography (HPLC) instrument that was equipped with an Aminex HPX-87 (BioRad, Hercules, CA, USA) column. The TOC content was determined using a TOC meter. The concentration of the PS was analyzed with an iodometric titration method, according to the procedure described in ref [6]. The amount of leached iron was determined by an analysis of the Fe ion concentration in the aqueous phase. Spectrophotometry at λ = 510 nm, after adding 1,10-phenanthroline to form an iron–phenanthroline complex, was used. Finally, the stability and reusability of the catalyst were evaluated.

## 3. Results and Discussion

### 3.1. Physicochemical and Structure Properties

**Figure 2:**
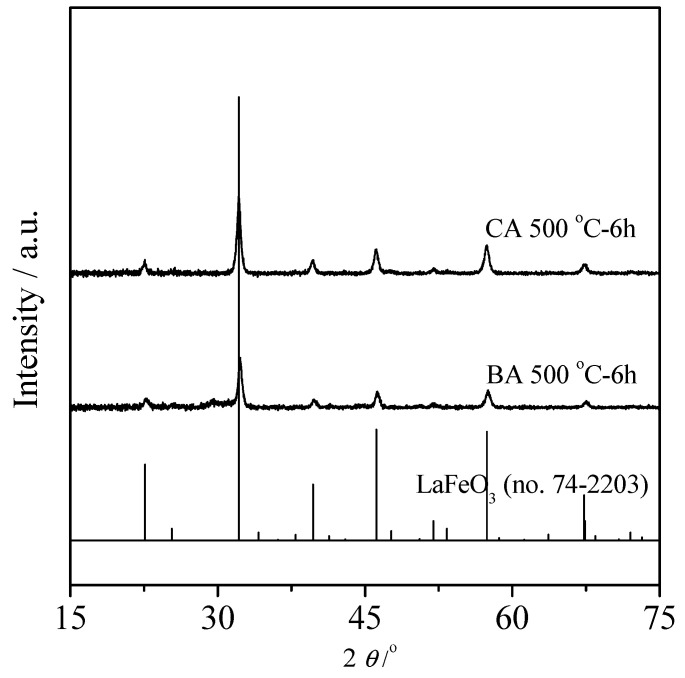
XRD results for LaFeO_3_ samples prepared by the BA and CA routes. Bottom of the figure: vertical lines for reference LaFeO_3_ (JCPDS card 74-2203).

The samples that were prepared by non-aqueous benzyl alcohol routes (BA) and citrate complexation routes (CA) showed reflections that were characteristic of the perovskite phase (Figure 2), being in perfect agreement with the orthorhombic structure of LaFeO_3_ (JCPDS card 74-2203). A small, broad, and poorly defined reflection of 28.220° (101) was visible on the baseline of the BA sample pattern. This reflection was identified in the La(OH)_3_ external phase, according to the JCPDS reference JCPDS No. 36-1481. The larger FWHM of the XRD peaks observed for the BA sample show that this material presented a lower crystal size than the CA sample at the iso-crystallization temperature (18.4 nm for BA vs. 22.3 nm for CA): an 18% lower crystal size was measured for the BA. These results confirm that the BA route was adapted for the production of crystalline-mixed oxide nanoparticles, starting from an organic–inorganic hybrid precursor [50]. A larger specific surface area (32.5 m^2^ g^−1^) was also obtained for the BA sample, which was much higher than the value for the LaFeO_3_ sample that was obtained by the CA route (15.3 m^2^ g^−1^), with a 112% higher SSA.

Starting from the value of the average crystal domain size obtained from the XRD, it is possible to calculate a theoretical surface area, assuming a cubic-shaped crystal and no contact between the adjacent elementary particles. Then, a ratio R between the experimental specific surface area and the theoretical surface area (*R* = S_BET_/S_th_, Table 1) gives an indication of the agglomeration degree of the elementary crystals in the different materials.

Then, when *R* = 1, the developed surface area is equal to the theoretical surface area, which characterizes a poorly agglomerated material. When *R* approaches 0, meaning that the theoretical surface area is larger than the experimental specific surface area, dense and poorly porous agglomerates are occured in the material [51,52]. A comparison between the *R* ratios obtained for the two samples showed that the BA sample presented a significantly higher ratio compared to that of the CA sample. This result indicates that the BA route prevented the potential agglomeration of the perovskite, leading to a greater accessible surface area.

**Figure 3:**
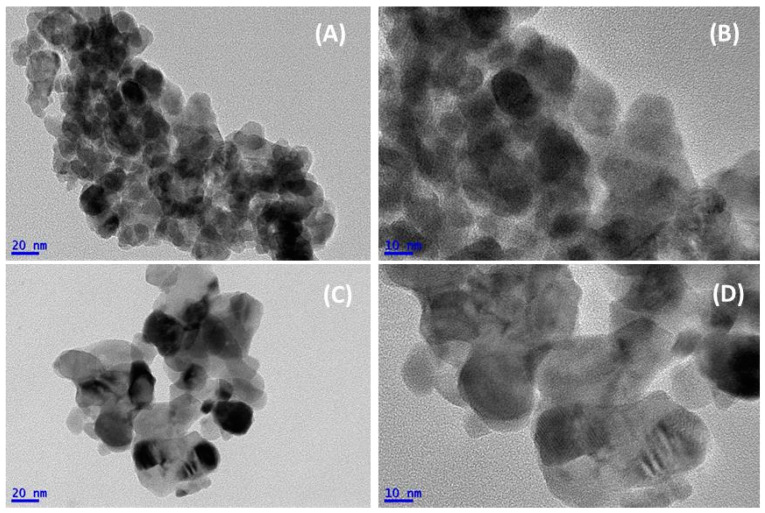
TEM images of BA catalyst (**A**,**B**), and CA catalyst (**C**,**D**) samples, after thermal stabilization at 500 °C.

The morphologies of the BA and CA LaFeO_3_ samples were investigated by TEM (Figure 3).

Based on the images in Figure 3A,B, the LaFeO_3_ sample that was synthesized via the BA route was composed of almost spherical LaFeO_3_ nanoparticles, with diameters ranging from 10 to 30 nm. These observed sizes were consistent with the average crystal domain sizes determined by the XRD (18.4 nm). The elementary particles formed small agglomerates, in which the presence of contrasted zones evidenced the formation of internal porosity. Slight differences were observed for the CA sample. While the low-magnification images evidenced the formation of agglomerates of more comparable sizes than those for the BA sample (Figure 3C), the high-magnification observation evidenced the agglomeration of elementary particles of sizes between 10 and 80 nm (Figure 3D). The particles showed inhomogeneity in their sizes and shapes. This morphology, mostly that of the larger average particle size, was at the origin of the lowest SSA (15.3 m^2^ g^−1^).

### 3.2. Process Configuration Effect on the Degradation of the TC Degradation

**Figure 4:**
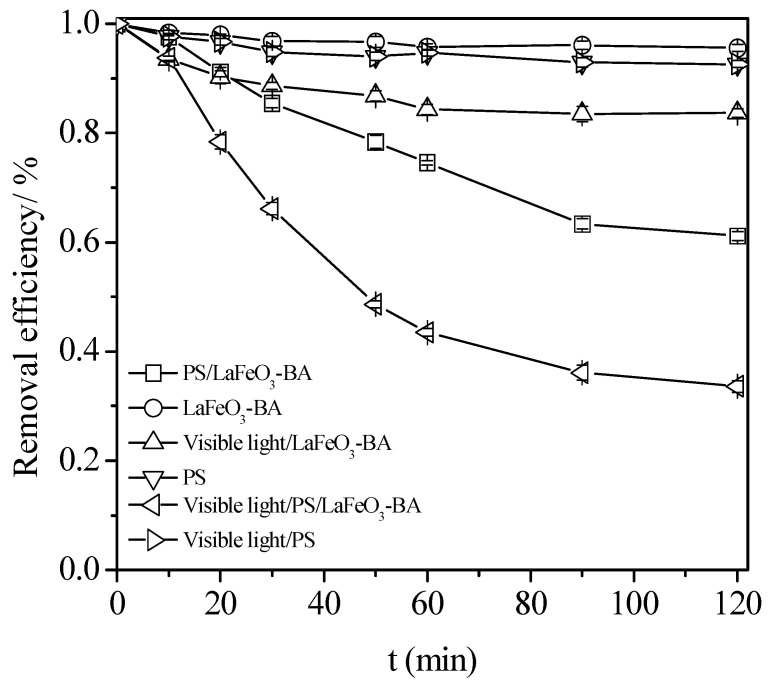
Process combination effect on the tetracycline degradation. Conditions: C_0_ = 100 mg L^−1^, [BA] = 0.5 g L^−1^, [PS] = 50 mM, and pH_0_ = 3.7.

Different configurations of the process were tested to evaluate their respective efficiencies in the TC degradation reaction (Figure 4):

- The PS alone (single process);
- The catalyst alone (single process);
- A combination of visible light/PS (dual process);
- A combination of PS/BA (dual process);
- A combination of visible light/BA (dual process);

For the combination of visible light/PS/BA (ternary process), the quantum yield was estimated to examine the photodegradation efficiency [53], which was found to be 2.3 × 10^−2^.

The photo stability of the tetracycline was also assessed. In order to explore the individual contribution of the photo-catalysis to the removal of the TC, the TC solution was irradiated by visible light alone until the equilibrium concentration of the TC, [TC]_0_, was obtained. A 15.5% decrease in the TC concentration was obtained by photolysis, given a [TC]_0_ value of 98.9 mg L^−1^ (corrected concentration). The retaining of more than 80% of the initial concentration evidenced the resistance of the TC toward to photo-catalytic degradation under visible irradiation alone.

In the single processes (PS or catalyst alone), the TC chemical degradation rate remained very slow under the selected operating conditions (22 °C, pH = 3.7). At the end of the reaction time, i.e., t = 120 min, the remaining concentration of the TC stayed above 92% of the [TC]_0_ value (Figure 4). The experiments realized with only the BA catalyst added to the solution also demonstrated a very limited efficiency, with the value of the TC concentration’s decrease remaining below 20%. Such an abatement (<10%) then demonstrates the limited ability of the catalyst surface for the simple adsorption of the organic pollutant.

Among the three configurations of the dual processes, the least efficient was the visible light/BA configuration, with an abatement that was limited to 14%, which is not surprising considering that, in this configuration, no efficient oxidant was added. Then, a slightly better efficiency was reached with the dual visible light/PS configuration, with a TC concentration abatement of 30% that was obtained after 120 min. However, this result shows that the process was poorly efficient when considering the stoichiometry of the reaction, being in excess of the PS (molar ratio PS/TC = 208.3) to afford the complete degradation of the pollutant. The best results were obtained during the last dual process, the coupling of PS and BA without light irradiation: a TC concentration abatement of 39% was obtained after 120 min. These results clearly indicate that the PS could be activated by the LaFeO_3_ to generate active radical oxidants.

When perovskites are irradiated with visible light energy that is equivalent to their band gap energy, the generation of photo-generated electrons and holes takes place. These photo-generated electrons and holes then participate in series of reactions and lead to the generation of active radical species (^•^OH), as shown below in Equations (9)–(13).

LaFeO_3_ + *hv* → LaFeO_3_ (e^−^ + h^+^) (9)

e^−^ + O_2_ → O_2_^•−^ (10)

O_2_^•−^ + H^+^ → HO_2_^•^ (11)

2e^−^ + HO_2_^•^ + H^+^ → ^•^OH + OH^−^ (12)

h^+^ + OH^−^ → ^•^OH (13)

h^+^ + H_2_O → H^+^ + ^•^OH (14)

Additionally, these photo-generated electrons and holes (h^+^) can undergo reactions with PS to enhance the production of SO_4_^•−^, as shown below in Equations (14) and (15):

e^−^ + S_2_O_8_^2−^ → SO_4_^•−^ + HO^−^ (15)

h^+^ + HO^−^ → ^•^OH (16)

Besides the role of photocatalysis, the activation of PS on the iron oxide surface induces the formation of radicals, including SO_4_^•−^ (Equations (17)–(19)) via a reaction on the catalyst surface [34], which will thereafter react with the TC. From a mechanistic point of view, ref. [35] demonstrated that the formation of these radicals involves an Fe^3+^/Fe^2+^ redox cycle, according to the following equations:

Fe^2+^ + HSO_5_^−^ → Fe^3+^ +SO_4_^•−^ + HO^−^ (17)

Fe^2+^ + HSO_5_^−^ → Fe^3+^ + SO_4_^2−^ + HO^•^ (18)

Fe^3+^ + HSO_5_^−^ → Fe^2+^ + SO_5_^•−^ + H^+^ (19)

Thereafter, the formed radicals (sulfate and/or hydroxyl) can attack the TC molecules and the TC molecules can be directly oxidized by the ^•^OH and SO_4_^•−^, thus generating by-products. These detected intermediates are then, to some extent, further mineralized to CO_2_ (Equation (20)).

^•^OH/SO_4_^•−^ + TC ↔intermediates ↔ CO_2_ + H_2_O + SO_4_^2−^ (20)

For the ternary process, when compared to the dual conditions, the degradation efficiency largely increased upon the visible irradiation being added to the coupled PS/BA. Indeed, the apparent constant rate value, *k_app_* (calculated assuming a pseudo-first-order reaction, list the different hypotheses), that was measured for the visible light/PS/BA was 0.0218 min^−1^, which was more than 6.8 times than the sum of the *k_app_* obtained for the visible light/PS (0.0032 min^−1^) and PS/LaFeO_3_ (0.0050 min^−1^). Especially compared to LaFeO_3_-CA (LaFeO_3_ syntheses by a citric acid complexation route), the pseudo-first-order reaction rate constant increased by 1.8 times. This indicated the synergistic effect of coupling the three conditions on the TC degradation rate. Furthermore, we evaluated the decomposition of the added PS that took place during the reaction. The utilization degree of the PS was 63% in the ternary visible light/PS/BA process, while it was only 21% for the dark dual PS/BA process. TC can absorb visible light energy into the excited state, facilitating an electron transfer to the Fe^3+^ surface site and resulting in PS decomposition into SO_4_^•−^ radicals, with an associated reduction in the iron surface site that is available on the catalyst surface, as proposed the above in Equations (16)–(18).

**Figure 5:**
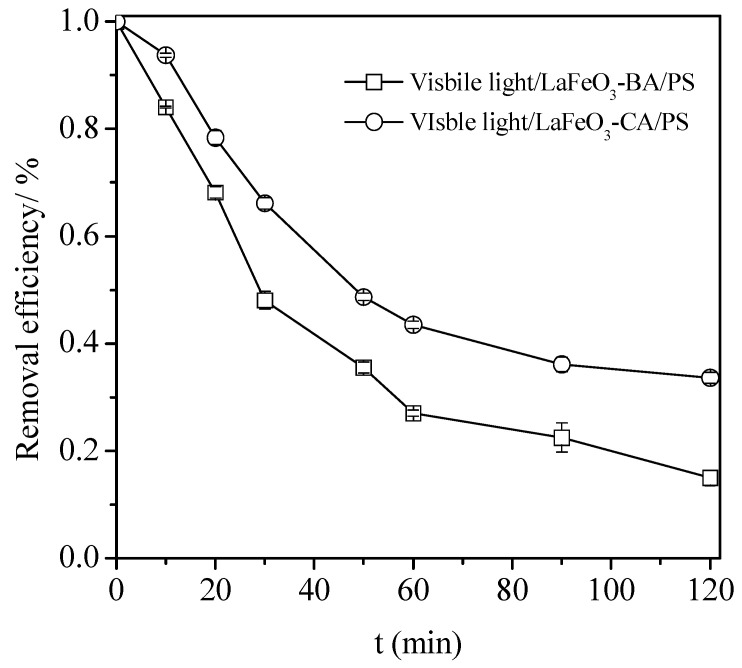
Impact of the preparation method on the catalyst efficiency in the visible light/PS/catalyst process for the tetracycline degradation. Conditions: C_0_ = 100 mg L^−1^, catalyst (BA or CA) = 0.5 g L^−1^, [PS] = 50 mmol L^−1^, and pH_0_ = 3.7.

In order to confirm this mechanism (surface activated/dependent or not), the CA and BA catalysts were tested under comparable conditions (Figure 5). While a k_app_ of 0.0218 min^−1^ was obtained with the BA catalyst, a value of 0.0122 min^−1^ was obtained with the CA catalyst.

This result demonstrated the beneficial effect of using a BA catalyst instead of a CA catalyst. Considering the surface-activated mechanism for oxidant radical formation, both the surface area (being higher for the BA, Table 1) and the Fe^3+^ surface site’s reducibility (also being higher for the BA) were expected to be at the origin of the highest performances obtained with the BA catalyst. TPR experiments indeed demonstrated an activation of the Fe^3+^ surface site with the BA at a temperature of approx. 400 °C below the temperature that was obtained for the CA, which was already obtained by our previous results on the oxygen mobility/reducibility of iron-based perovskites produced by a benzyl alcohol route [44].

### 3.3. Operating Parameter Impact on the Process Global Efficiency

#### 3.3.1. Catalyst Dosage and Oxidant Concentration Effect

**Figure 6:**
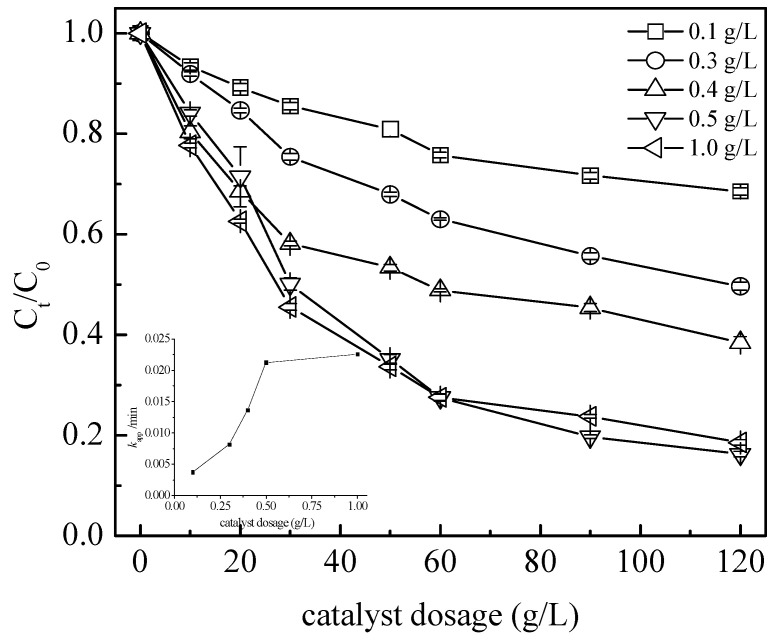
Effect of the BA catalyst dosage on the TC degradation. Conditions: C_0_ = 100 mg L^−1^, [PS] = 50 mM, and pH_0_ = 3.7.

The effect of the concentration of the BA on the TC degradation efficiency was also investigated (Figure 6). It was found that the TC removal efficiency and k_app_ increased with an increase in the catalyst loading when it increased from 0.1 g L^−1^ to 0.5 g L^−1^. At a BA loading of 0.5 g L^−1^, an 83.9% abatement of the [TC]_0_ was obtained after 120 min, corresponding to a k_app_ of 0.0218 min^−1^. A further increase of catalysts loading (BA) did not improve the TC abatement (84%) after 120 min. 

**Figure 7:**
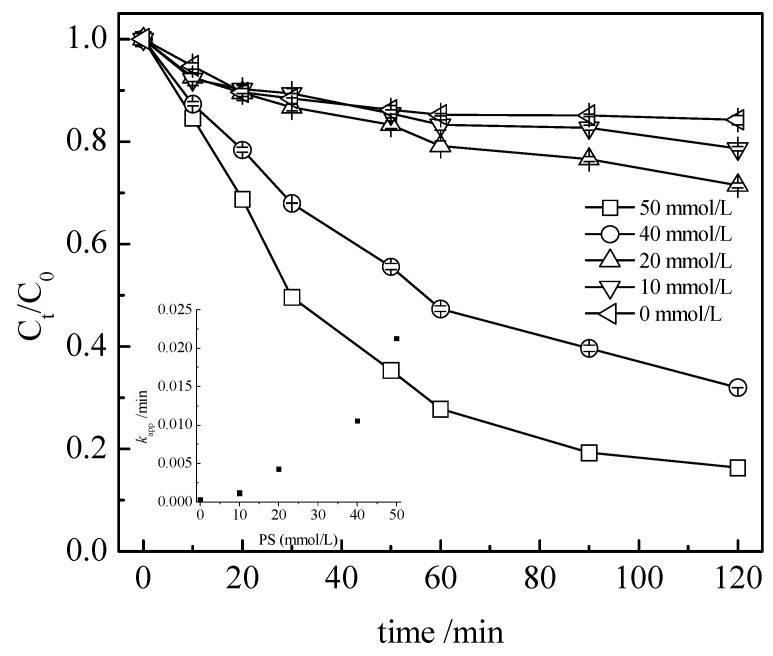
Effect of the sodium persulfate concentration on the tetracycline degradation. Conditions: C_0_ = 100 mg L^−1^, [BA] = 0.5 g L^−1^, and pH_0_ = 3.7.

The effects of the oxidants and PS concentrations on the degradation of the TC are presented in Figure 7 and also show that an increase in the oxidant concentration is beneficial to the TC abatement.

In this last case, the k_app_ value was linearly dependent on the PS concentration. Then, in the interval of the optimization selected, the catalyst loading and initial PS concentration values that led to the highest TC abatement, at a pH of 3.7, were 0.5 g L^−1^ and 50 mmol L^−1^, respectively.

#### 3.3.2. pH Value Effect

**Figure 8:**
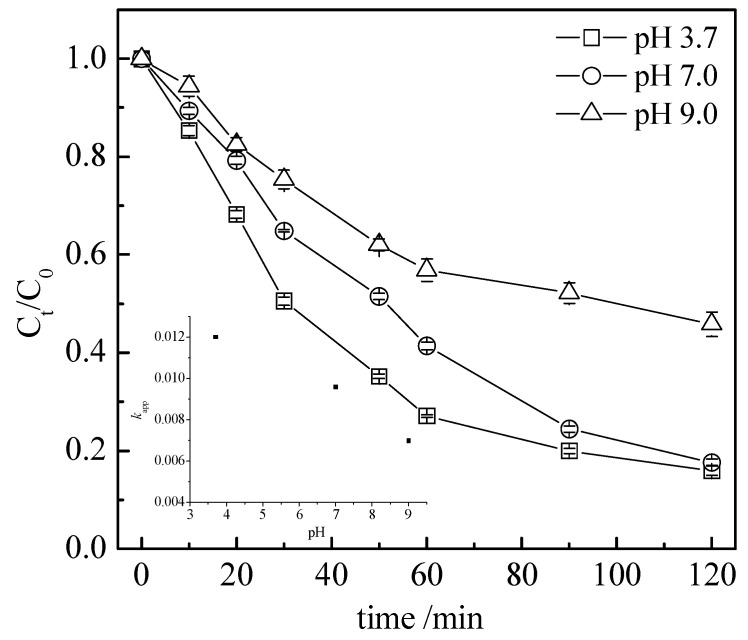
Effect of reaction medium initial pH on the tetracycline degradation. Conditions: C_0_ = 100 mg L^−1^, [BA] = 0.5 g L^−1^, and [PS] = 50 mmol L^−1^.

To investigate the pH effect on the degradation of the tetracycline, three different values of the initial pH (3.7, 7.0, and 9.0) were tested under acidic, neutral, and basic conditions. The results presented in Figure 8 show that the calculated TC removal efficiency and *k*_app_, assuming a first-order reaction rate, decreased accordingly with an increase in the pH value.

When compared to the value obtained at pH = 3.5 (*k_app_* = 0.012 min^−1^), the value of the *k_app_* decreased by 20% at pH = 7.0 to 0.0096 min^−1^. While the *k_app_* decreased significantly, the final TC abatement value was 82.3% after 120 min, which was very close to the value obtained at pH = 3.5, i.e., 83.9%. From a mechanism point of view, carboxyl acids (such as acetic acid) are produced during the reaction. Therefore, when the TC is degraded, the production of acidic intermediates induces a pH value decrease in the reaction medium when the reaction is performed without a buffer solution (such as in our case). This explains why, with a reaction starting at pH = 3.7, a final pH value of 2.6 was obtained after 120 min of the reaction. In the case of an initial pH of 7.0, the final value obtained was 2.7. Then, starting from 3.7 or 7.0, the final pH values of the reaction were comparable, explaining why comparable TC abatements were obtained in the two cases. The lowest value of the *k_app_*, 0.0073 min^−1^, was obtained when the initial pH value was increased to 9.0 and the TC abatement reached only 53%, a value far below the 82.3–83.9% obtained at the pHs of 3.7 and 7.0. In the case of an initial pH of 9.0, the pH value obtained at the end of the reaction was 4.2. These results demonstrate that the reaction was far more efficient in acidic conditions. However, the choice to perform the reaction without any buffer (such as NaHCO_3_/KHSO_4_ or Na_2_HPO_4_/KH_2_PO_4_) originated from the fact that buffer ions may act as scavengers of radicals such as SO_4_^•−^, as demonstrated in Equations (21) and (22) [44]:

SO_4_^•−^ + HCO_3−_ → SO_4_^2−^ + HCO_3_ (21)

HCO_3_ + H^+^ ⇋ CO_3_^2−^ (22)

### 3.4. Catalyst Stability Test

**Figure 9:**
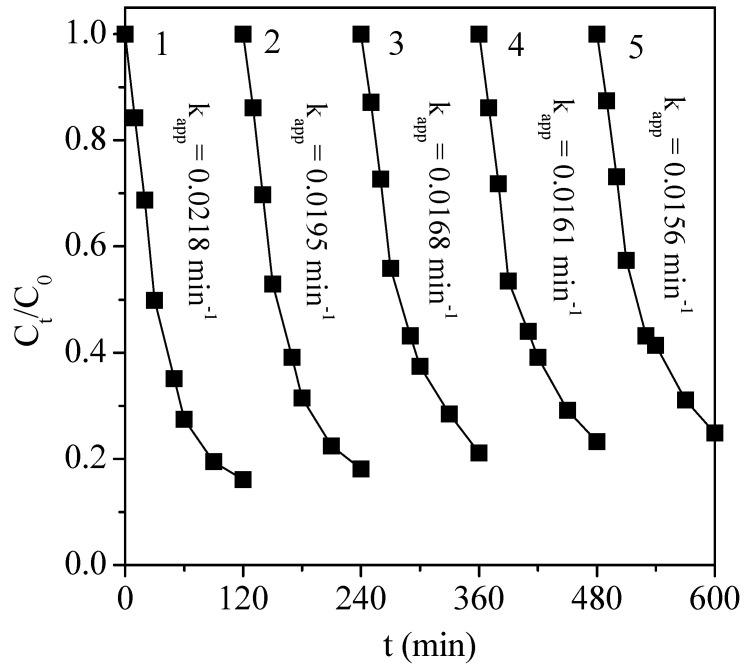
Evolution of the BA catalyst activity with the reaction cycles of TC degradation under visible light/PS/catalyst coupled process. Conditions: C_0_ = 100 mg L^−1^, [PS] = 50 mmol L^−1^, [BA] = 0.5 g L^−1^, and pH_0_ = 3.7.

The BA catalyst stability was investigated by the means of recycling experiments: the BA catalyst was repeatedly used in five successive batch experiments, as shown in Figure 9. After each run, the BA catalyst in the reaction solution was centrifuged, recovered, and dried at 100 °C for 12 h. The dried powder was directly used for the next reaction cycle, applying exactly similar conditions to those for the first run.

As can be observed in Figure 9, the BA was capable of being reutilized for at least five cycles, and the reused catalyst retained an acceptable catalytic activity. After the third cycle, the total removal efficiency of the TC was 83.9% at the first cycle. At the fifth cycle, the TC abatement always reached 79.3%. At this fifth cycle, the TC removal efficiency and the leached iron concentration were also evaluated. In addition, we also tested the TOC value. The result found that the TOC removal efficiency was 54.3%, which was an excellent level of mineralization considering the difficulty of degrading TC molecules. Furthermore, the leaching of Fe and La from LaFeO_3_ was also evaluated, quantifying the dissolved Fe and La in the reaction medium by ICP-OES: [La] = 0.03 mg L^−1^ and [Fe] = 0.02 mg L^−1^. This corresponded to a very limited leaching of the elements from the catalyst, in accordance with the maintenance of an excellent abatement during the five successive cycles at pH 3.7. To validate the heterogeneous character of the reaction, the reaction was performed with iron and lanthanum, which were added by the dissolution of nitrate precursors at the same concentrations as those obtained in the final reaction medium. A TC abatement of 3.81% was obtained after 120 min, which can be considered negligible in comparison to the 83.9% obtained in the presence of the catalyst. Then, this result confirmed the absence of a contribution of the dissolved species (either Fe or La) to the efficiency measured. It also allowed for the conclusion of a heterogeneous, mediated reaction, in which the nano-LaFeO_3_ behaved as an efficient and stable system when coupled with persulfate and visible irradiation.

### 3.5. Toxicity Evolution

Even though 83.9% of the TC was converted in the visible light/LaFeO_3_-BA/PS process after 120 min, the TOC conversion of the TC was only 54.3%. In this case, the variation in the acute toxicity was evaluated by a 48 h immobilization assay with *D. magna*. A 60% mortality rate of the *D. magna* was reached with a raw TC solution. After a 120 min reaction in the visible light/LaFeO_3_-BA/PS system, the toxicity reached a maximum value and 100% of the *D. magna* was immobilized, indicating that the formation of primary degradation by-products had a considerably higher toxicity than the TC. When the reaction time was extended to 240 min, the inhabitation percentage decayed, i.e., the mortality rate was only 45%. Therefore, prolonging the reaction time to reduce its acute toxicity is necessary for the TC treatment.

## 4. Conclusions

Tetracycline in an aqueous solution was efficiently degraded by a visible light/heterogeneous LaFeO_3_/persulfate process. The use of nano-LaFeO_3_ perovskite and visible LED lamps provided sensible advantages, including: (i) a low cost for the heterogeneous catalyst, (ii) convenient operation conditions (ambient temperature and no pressure), and (iii) a low power consumption (LED lamps irradiation). The properties of the nano-LaFeO_3_, especially its surface area, impact the efficiency of the global process. The other parameters influencing the final tetracycline degradation degree are: the catalyst loading, the persulfate loading, and the reaction medium pH. Satisfying the stability of the catalyst is obtained upon the reaction cycling, with a very limited leaching of the La and Fe. Nano-LaFeO_3_ can be considered to be an efficient heterogeneous catalyst, allowing for the use of persulfate as an oxidant to replace the more classically used H_2_O_2_, in view of the pollutant degradation in water.

## Figures and Tables

**Table 1:** Selected physico-chemical characteristics obtained for BA and CA catalysts.

Samples	BA Sample	CA Sample
Chemical composition	La_x_Fe_y_O_3±δ_	La_x_Fe_y_O_3±δ_
*D_XRD_*/^a^ nm	18.4	22.3
*S_th_*/^b^ m^2^ g^−1^	49.2	36.9
*S_BET_*/^c^ m^2^ g^−1^	32.5	15.3
*R*/^d^ -	0.66	0.41

^a^, *D_XRD_* is the crystal domain size determined using the Scherrer equation after correction of the FWHM for instrumental broadening; ^b^, *S_th_* is the theoretical surface area calculated from the surface area developed by a cubic particle volume of separated single crystal grains of *D_XRD_* size, assuming no contact between adjacent particles (no agglomeration); ^c^, *S_BET_* is the surface area determined using the B.E.T. equation; and ^d^, *R* = *S_BET_*/*S_th_*, characterize the degree of agglomeration in the material (*R* = 1, no agglomeration).

## Data Availability

Not applicable.
